# Hospital admissions in Alicante (Spain): a comparative analysis of foreign citizens from high-income countries, immigrants from low-income countries, and Spanish citizens

**DOI:** 10.1186/1472-6963-13-510

**Published:** 2013-12-09

**Authors:** José M Ramos, Eva M Navarrete-Muñoz, Hector Pinargote, Jaume Sastre, José M Seguí, María J Rugero

**Affiliations:** 1 Department of Internal Medicine, Hospital General Universitario de Alicante, Alicante, Spain; 2 Department of Clinical Medicine, Miguel Hernández University of Elche, Alicante, Spain; 3 Department of Public Health, Miguel Hernández University of Elche, Alicante, Spain; 4 CIBER en Epidemiología y Salud Pública (CIBERESP), Barcelona, Spain; 5 Department of Admissions and Documentation, Hospital General Universitario de Alicante, Alicante, Spain; 6 Department of Internal Medicine, Hospital Universitario de San Juan, Alicante, Spain; 7 Department of Admissions and Documentation, Hospital Universitario de San Juan, Alicante, Spain

**Keywords:** Immigrants, Foreigners, Public hospitals, Morbidity, Citizens, Low-income countries, High-income countries, Hospitalization

## Abstract

**Background:**

Over the last decade, the number of foreign citizens (FCs) in Spain has increased. There is no doubt that their health has become a relevant subject from the point of view of public healthcare. Our study aimed to describe hospital admission rates, diagnoses at hospital discharge, and mortality during hospital admissions in FCs from high-income countries (FCHICs), FCs from low-income countries (FCLICs), and autochthonous citizens (ACs).

**Methods:**

A cross-sectional study was performed at two public hospitals in the city of Alicante (Spain) and its surrounding area. Utilization rates were estimated. Multivariate analysis adjusting for age and sex was performed on hospital admission rates, diagnoses at hospital discharge, service of admission, and mortality during hospital admission in FCHICs and FCLICs compared with ACs (adjusted odds ratio [AOR] with 95% confidence intervals [CI]).

**Results:**

42,839 patients ≥15 years were discharged from the hospitals. The utilization rate was lower in FCs than ACs, whose crude rate ratio was 0.676 (95% CI: 0.656-0.696). FCHICs had more risk of being diagnosed at discharge in the categories of the circulatory system (AOR: 1.55; 95% CI: 1.35-1.77), neoplasms (AOR: 1.21; 95% CI: 1.03-1.42), and injury and poisoning (AOR: 1.33; 95% CI: 1.11-1.58). FCLICs had more risk of being diagnosed in the categories of pregnancy, childbirth & puerperium (AOR: 1.33; 95% CI: 1.29-1.59), and injury and poisoning (AOR: 1.19; 95% CI: 1.03-1.36), and less risk in the mental disorder category (AOR: 0.32; 95% CI: 0.22-0.45). The length of hospitalization (in days) was lower in FCLICs (median: 3; IQR: 2–6) than both ACs (median: 4; IQR: 2–8) and FCHICs (median: 4; IQR: 2–8) (p < 0.001). The mortality rates on admission of ACs, FCHICs, and FCLICs were 4.2%, 3.3%, and 1.3%, respectively, but after adjusting for age and sex, the mortality rate risks were similar in FCHICs and FCLICs.

**Conclusion:**

First, FCs utilized hospitalization less when compared with ACs. Second, the hospitalization profile for FCHICs was similar to ACs, with more problems in the circulatory system, and the hospitalization profile for FCLICs was different compared with ACs, with more admissions for pregnancy, childbirth & puerperium.

## Background

Immigration to Spain is relatively recent, constantly increasing, and is currently accepted as an intrinsic and widespread phenomenon of the nation’s demographics and social dynamics [1]. The number of foreign citizens (FCs) as a percentage of the population in Spain in the last decade has increased dramatically, from 2.9% in 1998 to 14.1% in 2011 [2]. In Spain, FCs come from many different areas, but generally they can be divided into two main groups: 1) FCs from high-income countries (FCHICs); and 2) FCs from low-income countries (FCLICs). Their impact on demographic changes to the health system can increase inequalities in health, but this phenomenon has yet to be evaluated.

Spain has a National Health System that is financed mainly by taxes, and it provides universal and free health coverage that includes primary, specialized, and hospital healthcare [3]. FCs may register in their municipality of residence to gain access to healthcare regardless of their legal status [4]. However, subsequent to the Spanish Health Law of September 2012, FCs without work permits have not been afforded universal health coverage except for special circumstances like pregnancies and emergency assistance [5]. Therefore, social inequalities towards FCs in the use of and access to health [3], and how they affect their health may leave them unprotected, outside the system, and could lead to higher spending on emergency services.

Several epidemiological studies have been performed in European countries exploring hospital utilization patterns by FCs. A Danish study [6] showed that hospital stay duration is longer for FCs than residents for some diagnoses, but shorter for others, although no overall effect was found. It has been suggested in the Netherlands [7] that FCs have an epidemiological profile similar to disadvantaged Dutch, although the prevalence of some infectious diseases and child mortality rates are higher among Turkish and Moroccan immigrants. An Italian study [8] showed that the hospitalization rate and length of hospitalization (in days) are lower for FCs than the Italian population. Another study from Italy reported that a main reason for hospitalization of foreign males in acute care was due to injuries, while among females, more than half of the admissions were for childbirth in acute or induced abortions [9]. In the Spanish context, several studies have been conducted on the health of FCs [10-12]. Nevertheless, there are few that have evaluated the impact of this migratory phenomenon on the use of hospital admissions in Spain’s healthcare system [13-17], or that have compared the differences or similitudes between FCHICs and FCLICs.

We consider it to be of interest to compare hospital admission rates, diagnoses at hospital discharge, service of admission at hospital discharge, and mortality during hospital admission between FCs and autochthonous citizens (ACs) before the Spanish Health Law of 2012 was enacted. Moreover, we compared the differences and similitudes between FCHICs and FCLICs in order to assess their needs and the impact on their health if these services are not offered.

## Methods

### Design and setting

A cross-sectional study was carried out at two public hospitals in Alicante (Spain) in 2011. The study area comprised the Hospital General Universitario de Alicante (HGUA) and the Hospital Universitario de Sant Joan d’Alacant (HUSJ). The former is located within the city of Alicante and the latter in Sant Joan d’Alacant (7 km north of Alicante). Individuals younger than 15 years of age were excluded from this study due to the difficulty of classifying their immigration status. These hospitals serve the city of Alicante and its surrounding areas, which were censused in January 2011 and numbered 487,546 inhabitants, of which 14.1% of this population was were FCs. Information about hospital discharges was obtained from the hospital information systems in 2011. This censused population uses either one of these two public hospitals, and some of these people, especially ACs, might use private hospitals. A total of 43,147 admissions attending in 2011 were included in the analysis; 308 of these came from countries whose origin was unknown, and so they were excluded from the study. The final analyses included 42,839 patients in the study.

### Variables

The present study defined FCs as people without Spanish citizenship. Immigrants can be granted Spanish citizenship only in very restricted circumstances. We identified the immigrants admitted and the immigrant population from their stated citizenship. The population was divided into ACs and FCs (FCHICs and FCLICs). FCHICs included patients born in 25 European Union countries, Switzerland, Iceland, Norway, the USA, Canada, Japan, and Australia. FCLICs included patients born elsewhere. The FCLICs were classified into the following groups: North Africa and the Middle East, Latin America, Eastern Europe, Sub-Saharan Africa, and Asia.

Hospital admissions data were obtained from the hospital information systems. The following data were collected each visit: demographic characteristics, nationality, diagnosis at discharge, unit of admission, destination at discharge (home, etc.), and length of stay. The diagnoses at discharge were performed according to the International Classification of Diseases, Ninth Revision, Clinical Modification (ICD-9-CM) [18] main groups of diagnoses. Hospitalization care episodes for discharges from the hospitals were classified into four large clinical specialties: medicine, surgery, gynecology, and traumatology.

The denominators of the rates for immigrants were based on data issued by the National Statistics Institute of Spain as of January 1, 2011. They include information on age (two age groups: 15–64 and >64 years), gender, and nationality, which allowed us to calculate rates for immigrants [19]. This database includes the total censused population living in the city of Alicante and its surrounding towns. Unfortunately, immigrants not counted in the town registries were not included, since estimating their number would have been very difficult. This database included all FCs, but did not differentiate between FCHICs and FCLICs. Therefore, the nationality variable is not disaggregated by age and sex, which hinders an adequate fit for the different variables.

For instance, crude utilization rates (cR) were calculated from patient origin (ACs or FCs) by age and sex as the number of hospitalizations from the defined group divided by the total residents in that group. The utilization rate was expressed as hospitalizations per 1,000 inhabitants per year. The crude rate ratios (cRR) of foreign citizens compared with ACs were calculated.

The Committee for Security of Information and Research at the Hospital General Universitario Alicante provided ethical approval for this study.

### Statistical analysis

Statistical analysis was performed with SPSS 19.0 (IBM, Chicago, IL, USA), and values of p < 0.05 were considered significant. The age distribution did not follow a normal distribution (Kolmogorov-Smirnov test with p < 0.05), so the age and length of hospitalization were therefore expressed as median and interquartile range (IQR). A descriptive analysis of a patient’s demographics, diagnoses, discharge, specialty, and outcome by ACs, FCHICs, and FCLICs was calculated. We compared the median age and length of hospitalization between ACs, FCHICs, and FCLICs with the Mann–Whitney U test. For bivariate comparison of proportions, either the Pearson χ2 method or the Fisher exact test method was applied. The measure of association was calculated by the odds ratio (OR) with a 95% confidence interval (CI). Multivariate logistic regression was used to estimate the ORs for diagnosis, discharge, specialty, and outcomes in ACs, FCHICs, and FCLICs after adjusting for sex and age (adjusted OR [AOR]).

## Results

In 2011, there were 408,763 residents ≥15 years old living in the study area, and of these, 14.1% were FCs. The percentage of female FCs is slightly lower than female ACs (52% vs 49%). Almost 91% of the FCs were between 15 and 64 years old compared with 80% of ACs. Of the total 42,839 admissions, 90% were ACs, 3% were FCHICs, and 7% FCLICs. Table 1 shows the ranking of the 40 FCs with the greatest numbers of patients admitted. The median age was 64 for both ACs (IQR: 43–77) and FCHICs (IQR 45–74), while for FCLICs it was 37 (IQR: 30–52) (p < 0.001). Females comprised 52%, 45%, and 66% of the ACs, FCHICs, and FCLICs, respectively (p < 0.001).

**Table 1:** Top 40 countries of the 4,219 foreign citizens (FCs) included in the present study

**High-income countries (N = 1,264)**				**Low-income countries (N = 2,955)**			
**Rk.**	**Country**	**N**	**%**	**Rk.**	**Country**	**N**	**%**
2	United Kingdom	432	10.2		**Latin America**	1,455	34.4
4	France	315	7.5	3	Colombia	329	11.1
8	Germany	104	2.5	4	Argentina	315	10.6
11	Italy	74	1.8	5	Ecuador	279	6.6
17	Belgium	62	1.5	10	Bolivia	82	1.9
19	Holland	55	1.3	13	Cuba	70	1.7
22	Switzerland	43	1.0	14	Paraguay	70	1.7
23	Poland	41	1.0	15	Uruguay	68	1.6
24	Portugal	38	0.9	18	Venezuela	56	1.3
30	Norway	25	0.6	25	Brazil	38	0.9
34	Sweden	19	0.4	27	Chile	34	0.8
36	Lithuania	18	0.4	28	Dominican Republic	32	0.8
40	Slovakia	11	0.3	29	Peru	31	0.7
				37	Mexico	17	0.4
				38	Nicaragua	12	0.3
				39	Honduras	12	0.3
					**North Africa**	695	16.4
				1	Morocco	478	11.3
				7	Algeria	200	4.7
					**Eastern Europe**	530	12.5
				6	Rumania	213	5.0
				9	Russia	96	2.3
				12	Bulgaria	73	1.7
				16	Ukraine	64	1.5
				32	Armenia	23	0.5
					**Sub-Saharan Africa**	165	3.9
				20	Nigeria	53	1.3
				26	Equatorial Guinea	35	0.8
				31	Senegal	24	0.6
					**Asia**	114	2.7
				20	China	53	1.3
				33	Pakistan	20	0.5
				35	India	19	0.4

Rk: ranking.

The cR for FCs versus ACs is shown in Table 2. The overall cR for the entire population studied was 105 hospitalizations per 1,000 inhabitants per year. The utilization rate was lower in FCs than ACs, with a cRR of 0.645 (95% CI: 0.626-0.696). The cRR of male FCs compared with that of male ACs was 0.501 (0.0478-0.526), and that of females was 0.799 (0.768-0.832). In all age ranges, the cRR for FCs was less than ACs.

**Table 2:** Population and crude utilization rate of adult hospitalizations for the total study sample: autochthonous (ACs), foreign citizens (FCs), and the crude rate ratio for FCs versus ACs in the total study sample and age and gender distribution

	**Total**	**ACs**	**FCs**	**FCs vs ACs cRR**	**95% CI**
Total					
Population	40,876	349,586	59,177	-	-
cR	105	111	75	0.676	(0.656; 0.696)
Sex					
Male					
Population	197,952	167,615	30,337		
cR	103	112	56	0.501	(0.478; 0.526)
Female					
Population	210,811	18,171	28,840		
cR	106	109	87	0.799	(0.768; 0.832)
Age					
15-64					
Population	331,032	277,233	53,689		
cR	70	72	61	0.842	(0.812; 0.873)
> 65					
Population	77,731	72,243	5,488		
cR	253	259	176	0.681	(0.642; 0.722)

CI: Confidence intervals.

cR: Crude utilization rate per 1,000 inhabitants per year.

cRR: Crude rate ratio.

Table 3 shows the main diagnosis groups in ACs, FCHICs, and FCLICs. After adjusting for age and sex, FCHICs had a greater risk of being discharged for circulatory system diseases (AOR: 1.55; 95% CI 1.35-1.77), neoplasms (AOR: 1.21; 95% CI: 1.03-1.42), and injury and poisoning (AOR: 1.33; 95% CI: 1.11-1.58).

**Table 3:** Number and percentage of hospital discharges by the ICD-9-CM main diagnosis group with the odds ratio (OR) and adjusted odds ratio (AOR) with a 95% confidence interval (CI) for foreign citizens from low-income countries (FCLICs) and high-income countries (FCHICs) compared with autochthonous citizens (ACs)

**Main diagnosis group**	**Origin of citizens**	**N**	**%**	**OR**	**(95% CI)**	**AOR**	**(95% CI)**
Circulatory system	ACs	7,179	18.6	1	-	1	-
	FCHICs	326	25.8	1.52	(1.34-1.73)	1.55	(1.35-1.77)
	FCLICs	261	5.5	0.44	(0.39-0.50)	0.89	(0.78-1.02)
Digestive system	ACs	4,861	12.6	1	-	1	-
	FCHICs	150	11.9	0.99	(0.72-1.01)	1.09	(0,92-1.21)
	FCLICs	340	11.5	0.90	(0.81-1.01)	1.01	(0.89-1.14)
Pregnancy, childbirth & puerperium	ACs	3,815	9.9	1	-	1	-
	FCHICs	103	8.1	0.98	(0.96-0.99)	0.20	(0.68-1.61)
	FCLICs	937	31.7	3.60	(3.35-3.86)	1.33	(1.29-1.59)
Neoplasms	ACs	4,393	11.4	1	-	1	-
	FCHICs	171	13.5	1.21	(1.03-1.42)	1.20	1.02-1.42)
	FCLICs	250	8.5	0.73	(0.64-0.83)	1.01	(0.88-1.16)
Respiratory system	ACs	4,069	10.5	1	-	1	-
	FCHICs	69	5.5	0.50	(0.39-0.63)	0.48	(0.37-0.61)
	FCLICs	189	6.4	0.59	(0.51-0.69)	0.89	(0,76-1.05)
Injury and poisoning	ACs	3,156	8.2	1	-	1	-
	FCHICs	135	10.7	1.33	(1.11-1.58)	1.33	(1.11-1.80)
	FCLICs	255	8.6	1.05	(0.93-1.19)	1.19	(1.03-1.36)
Genitourinary system	ACs	2,211	5.7	1	-	1	-
	FCHICs	62	4.9	0.99	(0.97-1.00)	0.93	(0.69-1,24)
	FCLICs	182	6.2	1.07	(0.93-1.24)	1.07	(0.91-1.26)
Nervous system & sense organs	ACs	2,221	5.8	1	-	1	-
	FCHICs	66	5.2	0.90	(0.71-1.16)	0.88	(0.68-1.13)
	FCLICs	137	4.6	0.80	(0.68-0.95)	0.85	(0.71-1.02)
Musculoskeletal system & connective tissue	ACs	1,558	4.0	1	-	1	-
	FCHICs	34	2.7	0.66	(0.47-0.93)	0.66	(0.47-0.93)
	FCLICs	95	3.2	0.88	(0.66-0.97)	0.81	(0.65-1.00)
Symptoms, signs & ill-defined conditions	ACs	1,345	3.5	1	-	1	-
	FCHICs	48	3.4	0.92	(0.69-1.21)	0.92	(0.69-1.25)
	FCLICs	76	2.6	0.74	(0.59-0.93)	0.79	(0.66-1.00)
Infectious & parasitic diseases	ACs	890	2.3	1	-	1	-
	FCHICs	29	2.3	0.99	(0.69-1.93)	0.99	(0.68-1.45)
	FCLICs	67	2.3	0.98	(0.78-1.24)	1.30	(1.00-1.69)
Endocrine, nutritional and metabolic diseases & immunity disorders	ACs	908	2.4	1	-	1	-
	FCHICs	19	1.5	0.64	(0.41-1.00)	0.64	(0.40-1.01)
	FCLICs	51	1.7	0.74	(0.56-0.97)	0.77	(0.57-1.03)
Mental disorders	ACs	877	2.3	1	-	1	-
	FCHICs	23	1.8	0.80	(0.53-1.20)	0.76	(0.50-1.16)
	FCLICs	34	1.2	0.52	(0.37-0.72)	0.32	(0.22-0.45)
Skin & subcutaneous tissue	ACs	563	1.5	1	-	1	-
	FCHICs	12	0.9	0.64	(0.36-1.15)	0.65	(0.36-1.159
	FCLICs	28	0.9	0.64	(0.44-0.94)	0.45	(0.34-0.73)
Blood & blood-forming organs	ACs	408	1.1	1	-	1	-
	FCHICs	13	1.0	0.97	(0.57-1.67)	0.97	(0.56-1.69)
	FCLICs	23	0.8	0.74	(0.50-1.11)	0.93	(0.60-1,43)
Congenital anomalies & conditions in the perinatal period	ACs	119	0.3	1	-	1	-
	FCHICs	3	0.2	0.77	(0.25-3.37)	0.77	(0.27-2.40)
	FCLICs	22	0.7	2.20	(1.50-3.23)	1.27	(0.79-2.04)

After adjusting for age and sex, FCLICs had a greater risk of diagnosis at discharge for the categories of pregnancy, childbirth & puerperium (AOR: 1.33; 95% CI: 1.29-1.59), and injury and poisoning (AOR: 1.19; 95% CI: 1.03-1.36), and less risk in the categories of mental disorders (AOR: 0.32; 95% CI: 0.22-0.45) and skin and subcutaneous tissue diseases (AOR: 0.45; 95% CI: 0.34-0.73).

Table 4 shows the absolute and relative numbers of discharges grouped by hospitalization specialty for ACs, FCHICs, and FCLICs. After adjusting for age and sex, FCHICs had a higher risk of discharge from surgery (AOR: 1.24; 95% CI: 1.01-1.23) and a lower risk from traumatology (AOR: 0.88; 95% CI: 0.59-0.95). Likewise, after adjusting for age and sex, FCLICs had a higher risk of discharge from gynecology (AOR: 1.46; 95% CI: 1.31-1.62) and a lower risk of discharge from medical specialties (AOR: 0.79; 95% CI: 0.73-0.87).

**Table 4:** Number and percentage of hospital discharges by discharge service with the odds ratio (OR) and adjusted odds ratio (AOR) with a 95% confidence interval (CI) for foreign citizens from low-income countries (FCLICs) and high-income countries (FCHICs) compared with autochthonous citizens (ACs)

	**Origin of citizens**	**N**	**%**	**OR**	**(95% CI)**	**AOR**	**(95% CI)**
Medicine	ACs	21,275	55.1	1	-	1	-
	FCHICs	709	56.1	1.04	(0.93-1.16)	1.02	(0.91-1.15)
	FCLICs	998	33.8	0.42	(0.38-0.45)	0.79	(0.73-0.87)
							
Surgery	ACs	9,293	24.1	1	-	1	-
	FCHICs	346	27.4	1.19	(1.05-1.35)	1.14	(1.01-1.23)
	FCLICs	682	23.1	0.95	(0.87-1.04)	1.01	(0.92-1.11)
							
Gynecology	ACs	5,053	13.1	1	-	1	-
	FCHICs	135	10.7	0.79	(0.63-0.95)	0.84	(0.68-1.04)
	FCLICs	1,087	36.8	3.87	(3.57-4.19)	1.46	(1.31-1.62)
							
Traumatology	ACs	2,999	7.8	1	-	1	-
	FCHICs	74	5.9	0.74	(0.58-0.94)	0.75	(0.59-0.95)
	FCLICs	188	6.4	0.81	(0.69-0.94)	0.88	(0.75-1.03)

Table 5 shows the lengths of hospitalization for the main diagnosis groups in ACs, FCHICs, and FCLICs. The length of hospitalization was shorter in FCLICs (median: 3; IQR: 2–6) than ACs (median: 4; IQR: 2–8) and FCHICs (median: 4; IQR: 2–8) (p < 0.001), especially in the categories of the circulatory system (p = 0.038), digestive system (p = 0.014), pregnancy, childbirth & puerperium (p = 0.003), respiratory system (p < 0.001), injury and poisoning (p = 0.003), genitourinary symptoms (p = 0.023), and symptoms, signs & ill-defined conditions (p = 0.011). However, the length of hospitalization was longer in FCHICs than ACs in the categories of the nervous system & sense organs (p = 0.044) and infectious & parasitic diseases (p = 0.015).

**Table 5:** Median and interquartile range of length of hospitalization of hospital discharges by the ICD-9-CM main diagnosis groups for foreign citizens from low-income countries (FCLICs) and high-income countries (FCHICs) compared with autochthonous citizens (ACs)

**Main diagnosis group**	**Origin of citizens**	**Median**	**(IQR)**	**p-value**
Total	ACs	4	(2–8)	
	FCHICs	4	(2–8)	0.89
	FCLICs	2	(3–6)	<0.001
Circulatory system	ACs	4	(2–8)	-
	FCHICs	4	(1–8)	0.67
	FCLICs	3	(1–8)	0.038
Digestive system	ACs	4	(2–8)	-
	FCHICs	3.5	(2–8)	0.32
	FCLICs	3	(1–8)	0.014
Pregnancy, childbirth & puerperium	ACs	3	(2–4)	-
	FCHICs	3	(2–4)	0.78
	FCLICs	3	(2–4)	0.003
Neoplasms	ACs	5	(3–10)	-
	FCHICs	6	(3–10)	0.28
	FCLICs	5	(3–9)	0.51
Respiratory system	ACs	5	(3–9)	-
	FCHICs	5	(2–8)	0.158
	FCLICs	4	(2–7)	<0.001
Injury and poisoning	ACs	5	(2–9)	-
	FCHICs	5	(2–9)	0.73
	FCLICs	3	(2–8)	0.003
Genitourinary system	ACs	4	(2–6)	1
	FCHICs	3	(2–5.5)	0.39
	FCLICs	3	(2–5)	0.023
Nervous system & sense organs	ACs	2	(1–5)	1
	FCHICs	2	(2–8)	0.044
	FCLICs	2	(1–5)	0.69
Musculoskeletal system & connective tissue	ACs	5	(2–7)	-
	FCHICs	5	(2–8.3)	0.31
	FCLICs	4	(2–7)	0.16
Symptoms, signs & ill-defined conditions	ACs	2	(1–5)	-
	FCHICs	2	(1–5.75)	0.30
	FCLICs	2	(1–3)	0.011
Infectious & parasitic diseases	ACs	6	(3–11)	-
	FCHICs	12	(5–16)	0.015
	FCLICs	7	(4–14)	0.30
Endocrine, nutritional and metabolic diseases & immunity disorders	ACs	4	(2–8)	-
	FCHICs	5	(4–7)	0.47
	FCLICs	4	(2–5)	0.24
Mental disorders	ACs	7	(4–13)	-
	FCHICs	5	(3–8)	0.16
	FCLICs	7	(3.10.5)	0.37
Skin & subcutaneous tissue	ACs	3	(2–7)	-
	FCHICs	2	(2–3)	0.41
	FCLICs	2	(1–4.8)	0.056
Blood & blood-forming organs	ACs	3	(5–9)	-
	FCHICs	2	(4.5-10)	0.37
	FCLICs	6	(3–10)	0.83
Congenital anomalies & conditions in the perinatal period	ACs	3	(2–3)	-
	FCHICs	2	(2-)	0.83
	FCLICs	2	(2–5)	0.91
Mortality	ACs	6	(2–14)	-
	FCHICs	8	(2.8-13)	0.67
	FCLICs	6	(1–13)	0.68

Table 6 shows the absolute and relative numbers of admissions from the emergency department, and discharges grouped by hospitalization outcome in ACs, FCHICs, and FCLICs. Admission from the emergency department was lower in FCHICs (58.9%) versus ACs (63.9%) (AOR: 0.84; 95% CI: 0.74-0.94), and higher in FCLICs (67.2%) (AOR: 1.22; 95% CI: 1.18-1.33). After adjusting for age and sex, FCHICs had a higher risk of transfer to other hospitals (AOR: 2.21; 95% CI: 1.67-2.92) and less risk of home hospitalization (AOR: 0.37; 95% CI: 0.18-0.79). After adjusting for age and sex, FCLICs had a higher risk of follow-up by specialists at home (AOR: 1.09; 95% CI: 1.01-1.19) and less risk of follow-up by general practitioners after hospital discharge (AOR: 0.91; 95% CI: 0.84-0.98). The mortality rates on admissions in ACs and FCHICs were 4.2% and 3.3%, significantly higher than FCLICs (1.3%) (p = 0.002). Nevertheless, after adjusting for age and sex, the higher mortality rate risk disappeared (AOR: 0.83; 95% CI: 0.59-1.16).

**Table 6:** Number and percentage of hospital discharges by origin of admission and outcome with the odds ratio (OR) and adjusted odds ratio (AOR) with a 95% confidence interval (CI) for foreign citizens from low-income countries (FCLICs) and high-income countries (FCHICs) compared with autochthonous citizens (ACs)

	**Origin of citizens**	**N**	**%**	**OR**	**(95% CI)**	**AOR**	**(95% CI)**
Admission from emergency department *	ACs (N = 34,745)	22,101	63.9	1		1	
	FCHICs (N = 1,140)	671	58.9	0.82	(0.73-0.92)	0.84	(0.74-0.94)
	FCLICs (N = 2,606)	1,750	67.2	1.17	(1.07-1.27)	1.22	(1.18-1.33)
Outcome							
At home and followed by general practitioner	ACs	23,230	60.2	1	-	1	-
	FCHICs	764	60.4	1.01	(0.90-1.13)	1.02	(0.91-1.15)
	FCLICs	1,703	57.6	0.90	(0.84-0.97)	0.91	(0.84-0.98)
							
At home and followed by specialist	ACs	11,431	29.6	1			
	FCHICs	353	27.9	0.92	(0.81-1.04)	0.91	(0.81-1.04)
	FCLICs	1,085	36.7	1.38	(1.28-1.50)	1.09	(1.01-1.19)
							
Home hospitalization	ACs	641	1.7	1		1.00	
	FCHICs	7	0.6	0.33	(0.16-0.70)	0.37	(0.18-0.79)
	FCLICs	17	0.6	0.34	(0.21-0.56)	1.05	(0.64-1.72)
							
Transfer to other hospitals	ACs	767	2.0	1	-	1	-
	FCHICs	55	4.4	2.24	(1.70-2.97)	2.21	(1.67-2.92)
	FCLICs	35	1.2	0.59	(0.42-0.83)	0.89	(0.63-1.28)
							
Death	ACs	1,638	4.2	1	-	1	-
	FCHICs	42	3.3	0.78	(0.57-1.06)	0.85	(0.62-1.59)
	FCLICs	37	1.3	0.29	(0.21-0.40)	0.83	(0.59-1.16)

*Only available for 38,491 cases.

## Discussion

Contrary to certain stereotypes that immigrants use health services excessively [20], the crude utilization rates across all age groups were lower in FCs than ACs. This result is consistent with previous reports of healthcare utilization by immigrant populations, like in primary care [21,22], specialized healthcare [23,24], emergency services [10-12], and hospitalization [13-17] in Spain and other European countries [6-9,25]. Our results corroborate the observation that the immigrant population has a lower hospitalization rate.

The countries of origin for the FCs were diverse, like the general immigrant distribution throughout Spain, and do not limit the external validity of the results to within Spain. Of the total number of hospitalizations at our hospitals in 2011, approximately 10% were FCs. These data were similar to those reported by Cots et al. [13] at the Hospital del Mar in Barcelona during 2002 and 2003, where 9.1% of hospital admissions were by immigrants. Salazar et al. [14], in the city of Valencia during 2002, reported that 18% of admissions were FCLICs. Ben Cheikh et al. [17] reported that 3.7% of hospitalizations at hospitals in the Aragon Community from 2004 to 2007 were FCs. In Italy, Cacciani et al. [8], in the Lazio region during 2000, reported that 2.2% of the discharges from hospitals were immigrants from less-developed countries.

In our study, approximately one-third of the FCs were FCHICs, while two-thirds were FCLICs. The prevalence of foreigners admitted to hospitals from FCHICs was higher than reported in other Spanish studies conducted with hospital admissions [13,17] or emergency services [10-12]. This data relates with our city on the Mediterranean coast, which is a residence for considerable numbers of European citizens, like reported in other Spanish cities along the Mediterranean coast [2].

First, we have found a higher proportion of emergency admissions among FCLICs than ACs. The FCLICs had a lower utilization of outpatient departments. For instance, they had a lower proportion of OPD admissions.

The profile of FCHICs presented in our study has not been thoroughly reported in other Spanish hospitalization research. This is because these individuals were either included within the AC group [13], were excluded [14], or were few in number and represented only 4.6% of all hospitalizations [17]. The profile of FCHICs was of adults with median ages similar to ACs, but with more males than females. Sixty percent of the patients came from the UK and France. After adjusting for age and sex, FCHICs had between 1.5, 1.3, and 1.2 times more risk of being discharged from the hospital compared with the native population for diseases related to the circulatory system (cardiovascular diseases), for injury and poisoning, and neoplasms, respectively. After adjusting for age and sex, FCHICs had 1.24 times more risk of being discharged from surgery and 0.77 times more risk from the service of traumatology. Transfers to other hospitals were more common and home hospitalization was less frequent than ACs. The length of hospitalization and mortality of FCHICs were similar to the native population.

FCLICs were younger than ACs and mainly female. This profile of young female patients has been reported in other studies of hospitalization [13-15,17] and admission into the emergency service [10-12] performed in Spain and Italy [8,9]. The main reasons for FCLIC hospitalizations were pregnancy, childbirth & puerperium, the digestive system, injury and poisoning, and neoplasms. In studies performed in Italy during 2000 and 2005 [8,9], the fifth reason for hospitalization was pregnancy, followed by injury and poisoning, then diseases of the digestive system. The prevalence of pregnancy, childbirth & puerperium in FCLICs in our study was 31%, similar to an Italian study (28.3%) [8]. In our study, injury and poisoning was responsible for 8.5%, less than the 12.6% of discharges from hospitals in Italian studies [8,9]. Discharge for digestive problems in our study was 11.5%, slightly more than in Spanish (9.1%) [17] and Italian studies (9.5%) [8].

Likewise, FCLICs had 1.33 times more risk of being discharged from clinical situations related to pregnancy, childbirth & puerperium, and 1.2 times from diseases of the group of injury and poisoning compared with the native population. Reproductive health for female FCLICs is emerging; the incidence of delivery and abortions is high in female FCLICs. Their hospitalization for pregnancy, childbirth & puerperium was found to be higher than the native population in all studies reported in Spain [13-15,17] and other FCHICs [8,9,26].

The higher prevalence of injury and poisoning in FCLICs compared with ACs suggests a greater vulnerability to injuries within this population. As such, Cacciani et al. [8] said the greater vulnerability to injuries might be related to poor living and working conditions. Surveys of occupational injuries conducted in Spain [27] and Italy [28] have suggested a higher risk of injuries for immigrants. Thereby, several studies conducted in Spain [29,30] and various European countries [31,32] have reported that migrant workers have higher rates of work-related accidents than ACs, especially in those undocumented foreign-born.

Discharges for mental disorders were 70% less in FCLICs. Our results agree with Ben Cheikh et al. [17], who report 0.3 times less risk of admission for mental problems in FCLICs. Nevertheless, in another study performed in Norway, the prevalence of mental disorders is the same or higher for immigrants than the autochthonous population [33]. In addition, an Italian study reported the same emergency room utilization for psychiatric problems in immigrants as well as Italian-born patients, while admissions to psychiatric wards were significantly less common in immigrants [34]. These results concur with our results. There may be barriers to the use of hospital resources for mental disorders in FCLICs due to less knowledge about these diseases, the stigmas of mental disorders, or a lack of referrals from primary care to the hospital.

In theory, FCLICs might be more infectious and carry more parasitic diseases than the native population because they come from countries at greater risk from these diseases [17,35]. In our study, the risk of discharge for infectious diseases in FCLICs was slightly higher than the native population after adjusting for age and sex (risk: 1.3 times).

On the other hand, FCLICs had 1.5 times more risk of discharge from obstetrics and gynecology. There was greater risk to these citizens in patients from all geographical areas. This is related to FCLIC females having a higher index of fecundity [14,17,36,37]. Therefore, discharges from obstetrics and gynecological services are higher than in native women. This pattern may be associated with social and cultural differences [37]. Nonetheless, in the UK, with more experience in attending ethnic minorities, the fertility trends in some UK ethnic groups have already fallen to about the level of the UK national average [38].

Likewise, FCLICs had 0.8 times less risk from medical specialties compared with ACs. This might be due to fewer medical diseases and more diseases treated by surgery in these patients. The length of hospitalization was shorter in FCLICs, probably due to greater risk of being admitted and discharged from the gynecology service and being younger than ACs. Mortality was lower in FCLICs; similar results have been reported in the Spanish study by Clots et al. [13]. However, in our study, after adjusting for age and sex, the risk disappeared.

Among this study’s limitations was the impossibility of analyzing the utilization rates of FCLICs and FCHICs adjusted for age and sex. This is because data for registered patients at Spain’s National Statistics Institute that are separated by age and sex are not available by country of origin. Moreover, the hospitalization registry does not include information on socioeconomic status, something that the literature has shown to be related to differences in healthcare access [11]. Another limitation was that we did not calculate the cost of hospitalization in ACs, FCLICs, or FCHICs, because the study’s objective was to describe the clinical profile of hospitalization but not the costs, and because there are several studies that have already examined this aspect in Spain [10-16]. Economic analysis provides a certain level of evidence that is useful for planning health services and interventions to improve healthcare in the health area [12].

## Conclusions

Some conclusions for health policy may be drawn from the results of this study. First, the lower utilization of hospitalization by FCs compared with ACs suggests that the population increase due to immigration does not translate directly into a corresponding increase in the number of hospitalizations in the area. Second, the hospitalization profile in FCHICs was close to ACs with a higher risk from the circulatory system, and the profile of hospitalization in FCLICs was different by age, sex, diagnoses, and discharging service. Finally, there was greater hospital utilization of obstetrics and gynecological services by FCLICs, suggesting that greater efforts should be made in this healthcare area.

## Consent

The patients given his/her oral consent to be included in the hospital information systems.

## Competing interests

The authors declare that they have no competing interests.

## Authors’ contributions

JMR made substantial contributions to the conception and design. JS and MJR participated in the acquisition of data. JMR, HP, JMS, and EMNM were involved in analysis and interpretation of data. JMR, HP, and EMVN were involved in drafting the manuscript. JMR, HP, JMS, JS, MJR, and EMNM revised the manuscript. All authors read and approved the final manuscript. All authors read and approved the final manuscript.

## Pre-publication history

The pre-publication history for this paper can be accessed here:

http://www.biomedcentral.com/1472-6963/13/510/prepub
